# *Mycobacterium massiliense* Induces Inflammatory Responses in Macrophages Through Toll-Like Receptor 2 and c-Jun N-Terminal Kinase

**DOI:** 10.1007/s10875-013-9978-y

**Published:** 2014-01-09

**Authors:** Tae Sung Kim, Yi Sak Kim, Heekyung Yoo, Young Kil Park, Eun-Kyeong Jo

**Affiliations:** 1 Department of Microbiology, College of Medicine, Chungnam National University, 6 Munhwa-dong, Jungku, Daejeon, 301-747 South Korea; 2 Infection Signaling Network Research Center, College of Medicine, Chungnam National University, Daejeon, 301-747 South Korea; 3 Department of Research and Development, Korean Institute of Tuberculosis, Osong Bio-Health Science Technopolis, Chungbuk, 363-954 South Korea

**Keywords:** *Mycobacterium massiliense*, Dectin-1, TLR, MyD88, ROS, NF-kB, TNF-α, IL-6

## Abstract

*Mycobacterium massiliense* (Mmass) is an emerging, rapidly growing mycobacterium (RGM) that belongs to the *M. abscessus* (Mabc) group, albeit clearly differentiated from Mabc. Compared with *M. tuberculosis*, a well-characterized human pathogen, the host innate immune response against Mmass infection is largely unknown. In this study, we show that Mmass robustly activates mRNA and protein expression of tumor necrosis factor (TNF)-*α* and interleukin (IL)-6 in murine bone marrow-derived macrophages (BMDMs). Toll-like receptor (TLR)-2 and myeloid differentiation primary response gene 88 (MyD88), but neither TLR4 nor Dectin-1, are involved in Mmass-induced TNF-α or IL-6 production in BMDMs. Mmass infection also activates the mitogen-activated protein kinase (MAPKs; c-Jun N-terminal kinase (JNK), ERK1/2 and p38 MAPK) pathway. Mmass-induced TNF-α and IL-6 production was dependent on JNK activation, while they were unaffected by either the ERK1/2 or p38 pathway in BMDMs. Additionally, intracellular reactive oxygen species (ROS), NADPH oxidase-2, and nuclear factor-κB are required for Mmass-induced proinflammatory cytokine generation in macrophages. Furthermore, the S morphotype of Mmass showed lower overall induction of pro-inflammatory (TNF-α, IL-6, and IL-1β) and anti-inflammatory (IL-10) cytokines than the R morphotype, suggesting fewer immunogenic characteristics for this clinical strain. Together, these results suggest that Mmass-induced activation of host proinflammatory cytokines is mediated through TLR2-dependent JNK and ROS signaling pathways.

## Introduction

Rapidly growing mycobacteria (RGM) are emerging human pathogens causing diverse non-tuberculous mycobacterial (NTM) diseases, especially opportunistic infections in immunocompromised patients [1]. In recent years, the numbers of RGM and newly identified NTMs have increased markedly in the clinical field due to improved genetic identification methods for mycobacterial species [2–4]. *Mycobacterium massiliense* (Mmass) is a recently isolated RGM strain, which belongs to the *M. abscessus* (Mabc) group, because it has high similarity in genetic sequences with Mabc [5]. However, they are clinically and genetically separate species. In clinical aspects, treatment outcomes with clarithromycin therapy are much better in patients infected with Mmass than in those infected with Mabc [6].

A fine balance in inflammatory responses triggered by mycobacterial infection is important in mounting host protective immune responses and the establishment of mature granulomas [7]. The proinflammatory cytokine tumor necrosis factor (TNF)-α is known to play a central role in the formation of granulomas, the sites of protection and pathology in tuberculosis [8–10]. Anti-TNF-α immunotherapy has been associated with increased risk of tuberculosis reactivation due in part to a decrease in antimicrobial activity and CD8+ effector memory T cell activation [11, 12]. These results are consistent with previous work showing that both TNF-α and interleukin (IL)-6 play a role in controlling *M. tuberculosis* (Mtb) bacterial infection, with significant impacts on survival in a mouse model of Mtb infection [13, 14]. In vivo analyses of Mabc infection further support a role for TNF-α as a critical mediator of bacterial inhibition in liver and spleens, controlling both hepatic inflammation and granuloma formation [15]; a more detailed examination of the cytokine profiles elicited during Mmass infection has not been performed.

Recent studies on Toll-like receptor (TLR) signaling during mycobacterial infection have revealed the molecular mechanisms by which mycobacteria and their ligands induce innate defense and proinflammatory responses [16, 17]. TLR-triggered innate immune responses are regulated by complex intracellular signaling cascades involving nuclear factor (NF)-κB and mitogen-activated protein kinase (MAPK) pathways [16, 18]. Both signaling pathways play key roles in the activation of antimicrobial responses and in the generation of effector molecules during mycobacterial infection [19]. Moreover, reactive oxygen species (ROS), derived primarily from NADPH oxidases (NOX), are important in shaping and controlling the key signaling network system redox-regulation-during infectious and inflammatory responses [20, 21]. Previous studies have also shown that Mtb triggers ROS generation, and ROS play a role in proinflammatory responses in macrophages [22]. However, the host innate immune responses against Mmass infection has remained largely unknown, compared with what is known about Mtb or other NTM infections, such as with Mabc.

In this study, we investigated the intracellular signaling pathways activated by Mmass infection in murine bone marrow-derived macrophages (BMDMs). First, we determined whether Mmass induced TNF-α and IL-6 production in BMDMs. We then examined the roles of TLR2, TLR4, MyD88, and Dectin-1 in Mmass-mediated TNF-α and IL-6 production in BMDMs. We further examined the activation of the MAPK pathway (c-Jun N-terminal kinase (JNK), ERK1/2 and p38 MAPK), NF-κB, and ROS by Mmass infection and the role by which MAPK and ROS regulate Mmass-induced proinflammatory responses in BMDMs. Finally, we examined the profiles of proinflammatory signaling activation in macrophages in response to infection with Mmass clinical strains, the R and S morphotypes.

## Materials and Methods

### Cultures of Mmass and Mabc

Mmass of type strain CIP 108297, two clinical smooth strains (KMRC00136-13008 and KMRC00136-13011) and two clinical rough strains (KMRC00136-13009 and KMRC00136-13012) were obtained from The Korean Institute of Tuberculosis (Osong, Korea). The Mabc type strain ATCC 19977 and all Mass strains were cultured as described previously [23]. All type-strain colonies exhibited smooth morphotypes. The mycobacteria were grown in Middlebrook 7H9 medium (Difco Laboratories, Detroit, MI, USA) with 10 % OADC supplement (BD Pharmingen, San Diego, CA, USA), 0.5 % glycerol, and 0.05 % Tween 80 (Sigma-Aldrich, St. Louis, MO, USA) at 37 °C. Mmass and Mabc were collected by centrifugation, homogenization, and filtration. Frozen bacteria were stored at −70 °C. Representative Mmass and Mabc vials were thawed and the numbers of colony forming units on Middlebrook 7H10 agar plates were counted.

### Mice and Ethics Statement

Mice with the C57BL/6 wild-type (WT) background were purchased from SAMTAKO BIO KOREA (Gyeonggi-do, Korea), C3H/HeN (TLR4 wild-type) and C3H/HeJ (TLR4-deficient) mice were purchased from The Jackson Laboratory (Bar Harbor, ME, USA). All mice were on the C57BL/6 background. TLR2, MyD88, and TRIF-knockout (KO) mice were kindly provided by Dr. S. Akria (Osaka University, Japan) and NOX2 KO mice were kindly provided by Dr. Bae (Iwha University, Korea). This study was approved by the Institutional Research and Ethics Committee at Chungnam National University. All animal procedures were conducted in accordance with the guidelines of the Korean Food and Drug Administration (KFDA).

### Culture of BMDMs and Cell Lines

BMDMs and Raw264.7 cell line were cultured as described previously [24]. The BMDMs (Lonza; Walkersville, MD, USA) were from mice sacrificed at 6-8 weeks of age. BMDMs were isolated from femurs of mice killed by cervical dislocation and cultured for 4 days in 20 μg/mL macrophage colony-stimulating factor (R&D Systems, Minneapolis, MN, USA) and supplement-containing culture medium (Dulbecco’s modified Eagle’s medium, 10 % heat-inactivated fetal bovine serum, 1 mM sodium pyruvate, 50 U/mL penicillin, and 50 μg/mL streptomycin). Murine macrophage RAW264.7 cells (ATCC TIB-71; Manassas, VA, USA) were grown in supplement-containing culture medium.

### Reagents

Lipopolysaccharides (LPS; *Escherichia coli* 0111:B4) and synthetic bacterial lipopeptide (Pam3Cys-Ser-Lys4-OH) were from InvivoGen (San Diego, CA, USA). N-acetylcysteine (NAC), diphenyleneiodonium (DPI), BAY11-7082 (BAY), caffeic acid phenethyl ester (CAPE), U0126, SB203580, and SP600125 were from Calbiochem (San Diego, CA, USA). 4,5-dihydroxy-1,3-benzene disulfonic acid disodium salt (Tiron) and dimethyl sulfoxide (DMSO; added to the cultures at 0.05 % (v/v) as a solvent control) were from Sigma-Aldrich. Phospho-SAPK/JNK (Thr183/Tyr185), phospho-ERK1/2 (Thr202/Tyr204), and phospho-p38 (Thr180/Tyr182) were from Cell Signaling Technology (Cell Signaling; Beverly, MA, USA). β-actin (I-19) was obtained from Santa Cruz Biotechnology (Santa Cruz, CA, USA).

### Western Blot Analysis and Enzyme-Linked Immunosorbent Assay (ELISA)

Western blot analysis and ELISA were conducted as described previously [23]. For Western blotting, whole protein extracts were denatured by boiling and resolved in 12 % acrylamide SDS-PAGE gels. Then, proteins were transferred to a polyvinyl difluoride membrane (Millipore, Boston, MA, USA). For Western blotting, primary antibodies were diluted at a ratio of 1:1000. The membranes were developed with ECL solution (Millipore) and were exposed to chemiluminescence film (Fujifilm, Japan).

For ELISA (BD PharMingen), infected cell supernatants were assessed for TNF-α, IL-6, IL-1β and IL-10 secretion using Duoset antibody pairs, according to the manufacturer’s protocol.

### Reverse Transcriptase-Polymerase Chain Reaction (RT-PCR) Analysis

For semi-quantitative RT-PCR analysis, total RNA was extracted from cells using TRIzol (Invitrogen, Carlsbad, CA, USA), as described previously [23]. Primer sequences were as follows: *TNFα* (forward: 5′-CGGACTCCGCAAAGTCTAAG-3′, reverse: 5′-ACGGCATGGATCTCAAAGAC-3′), *IL6* (forward: 5′-GGAAATTGGGGTAGGAAGGA-3′, reverse: 5′-CCGGAGAGGAGACTTCACAG-3′), *IL1B* (forward: 5′-CTCCATGAGCTTTGTACAAG-3′, reverse: 5′-TGCTGATGTACCAGTTGGGG-3′), *IL10* (forward: 5′-ATGCAGGACTTTAAGGGTTA-3′, reverse: 5′-ATTTCGGAGAGAGGTACAAA-3′), and *GAPDH* (forward: 5′-TGGCAAAGTGGAGATTGTTTG-3′, reverse: 5′-AAGATGGTGATGGGCTTCCC-3′). For TNFα, IL-6, IL-1β, IL-10 and GAPDH annealing was performed at 58 °C for 45 s. RT-PCR products were resolved in a 1.5 % agarose gel and visualized by staining with ethidium bromide.

### Transfection of Small Interfering RNA (siRNA) into Raw264.7 Cells

SiRNA transfection was performed as described previously [24]. Mouse dectin-1 siRNA (sc-63277; siDec-1) was purchased from Santa Cruz Biotechnology. Raw264.7 cells were transfected with 200 nM of scrambled siRNA or dectin-1-specific siRNA with the GenMute siRNA and DNA transfection reagent (SignaGen Laboratories, Ijamsville, MD, USA), according to the manufacturer’s protocol. Transfected cells were infected with Mmass or Mabc after then harvested for RT-PCR and ELISA.

### Generation and Transduction of Small Hairpin RNA (shRNA)

Lentivirus generation and transduction of shRNA was performed as described previously [23]. The lentiviral construct vector pLKO.1 and three packaging plasmids (pMDLg/pRRE, pRSV-Rev and pMD2.VSV-G) were from Open Biosystems (Huntsville, AL, USA) and target dectin-1 (TRCN0000066928) was from Sigma-Aldrich, supplied as glycerol stocks. Lentivirus generation was achieved by transfecting with Lipofectamine 2000 into HEK 293 T cells with pLKO puro.1 or target shRNA plasmid and the packaging plasmids. After 72 h, the virus-containing HEK 293 T cell culture supernatants were collected and filtered. Lentivirus determination was performed as described previously [23]. The lentivirus particles mixed with 8 μg/mL Polybrene (Sigma-Aldrich) and non-specific shRNA or dectin-1 shRNA into BMDMs, according to the manufacturer’s protocol. After transduction, the BMDMs were harvested and the target gene-silencing efficiency was examined by RT-PCR analysis.

### Measurement of Intracellular ROS

Intracellular ROS levels were measured as described previously [24]. Briefly, BMDMs were infected with Mmass and washed three times in phosphate-buffered saline (PBS) to remove extracellular bacteria. The cells were incubated with 10 μm dihydroethidium (DHE; Calbiochem) for 30 min and fixed with 4 % paraformaldehyde at room temperature followed by analysis with a FACSCanto II flow cytometer (Becton Dickinson, San Jose, CA, USA). Analysis of 30,000 events per sample was performed using the FlowJo software (Tree Star; Ashland, OR, USA).

### Immunofluorescence Microscopy of NF-κB p65 Translocation

Immunofluorescence analysis was performed as described previously [25]. Briefly, BMDMs were infected with Mmass after they were fixed with 4 % paraformaldehyde (for 10 min) and 0.25 % Triton X-100 (for 15 min) at RT. The cells were stained with primary antibody (rabbit anti-mouse NF-κB p65, 1:400, for 2 h; Santa Cruz) and secondary antibody (anti-rabbit AlexaFluor 488, 1:400, for 1 h; Invitrogen) at RT. Nuclei were stained with 1 μg/mL DAPI (Sigma-Aldrich). The slides were imaged using a Zeiss LSM510META confocal microscope (Zeiss, Germany).

### NF-κB Luciferase Reporter Assay

The NF-κB luciferase reporter assay was performed as described previously [25]. Briefly, cells were transduced with adenovirus harboring NF-κB-luciferase (Genetransfer Vector Core; Iowa City, IA, USA) for 36 h, and infected with Mmass for 6 h. Infected cells were washed three times in PBS, and then lysed in luciferase lysis buffer (Promega, Madison, WI, USA). A luciferase assay system (Promega) was used according to the manufacturer’s instructions.

### Statistical Analyses

All data are presented as mean values ± SD of three independent determinations. For statistical analyses, paired t-tests with Bonferroni adjustment or analysis of variance for multiple comparisons were performed. Differences were considered significant at P < 0.05.

## Results

### Mmass Induces Proinflammatory Responses in BMDMs via TLR2

TLRs are the best-characterized innate receptors in initiating inflammatory responses during Mtb infection [17]. Our previous studies showed that Mabc also induced innate immune response through TLR2 [26]. However, it was not known whether proinflammatory responses are induced by Mmass infection or how these are modulated in macrophages.

**Fig. 1:**
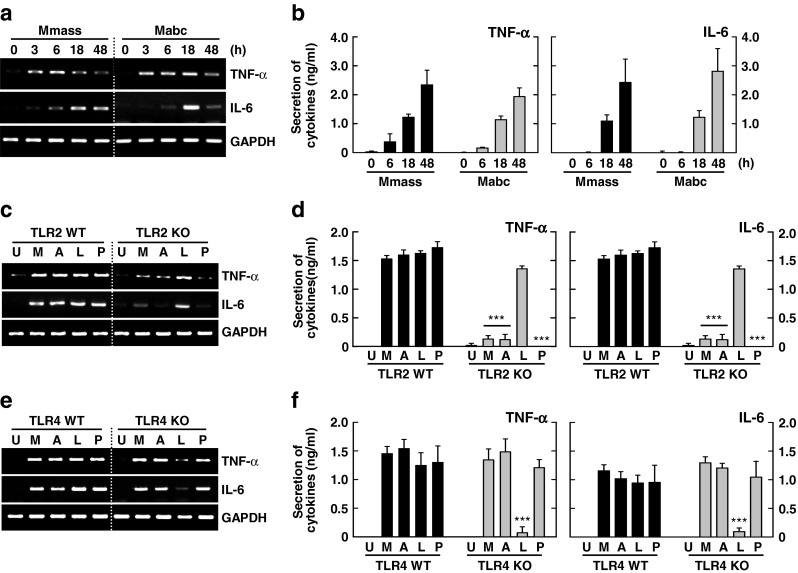
Mmass induces TNF-α and IL-6 production in a TLR2-dependent manner. **a** and **b** BMDMs were infected with Mmass (MOI = 3) or Mabc (MOI = 3) for the times indicated (0-48 h). **c**–**f** BMDMs from TLR2 WT and TLR2 KO BMDMs (**c** and **d**) or TLR4 WT and TLR4 KO BMDMs (**e** and **f**) were exposed to various stimulators; Mmass, Mabc, LPS (*L*; 100 ng/mL), or Pam3CSK4 (*P*; 100 ng/mL). **a**, **c**, and **e** Cell lysates were collected and then subjected to semiquantitative RT-PCR for *TNF-α* and *IL-6*. **b**, **d**, and **f** Infected supernatants were harvested and analyzed by ELISA for TNF-α and IL-6. Data shown are representative of at least three independent experiments (expressed as means ± SD). *** *p* < 0.001 (two-tailed Student’s *t*-test), relative to TLR2 WT (**d**) or TLR4 WT conditions (**f**). *U* uninfected, *M* Mmass, *A* Mabc

To examine this, we first measured and compared the time-dependent expression of mRNA and proteins of proinflammatory cytokines in BMDMs between infection with Mmass and Mabc. As shown in Fig. 1a and b, Mmass induced the expression of both mRNA and protein of TNF-α and IL-6 in BMDMs in a time-dependent manner. Mmass-induced proinflammatory cytokine production was comparable to that induced by Mabc (Fig. 1a, b). We next examined TNF-α and IL-6 mRNA and protein expression in BMDMs from WT or TLR2-KO mice, and TLR4-deficient C3H/HeJ or their control, TLR4-sufficient, C3H/HeN mice. As shown in Fig. 1c and d, Mmass-induced TNF-α and IL-6 mRNA and protein expression was significantly inhibited in TLR2-deficient BMDMs. In contrast, there was no difference in Mmass-induced TNF-α or IL-6 mRNA or protein expression in TLR4-deficient C3H/HeJ or C3H/HeN mice (Fig. 1e, f). These data suggest that TLR2, but not TLR4, is associated with Mmass-induced production of TNF-α and IL-6 in BMDMs.

### Mmass Induces a Proinflammatory Response Mediated Through MyD88, but not TRIF, in BMDMs

**Fig. 2:**
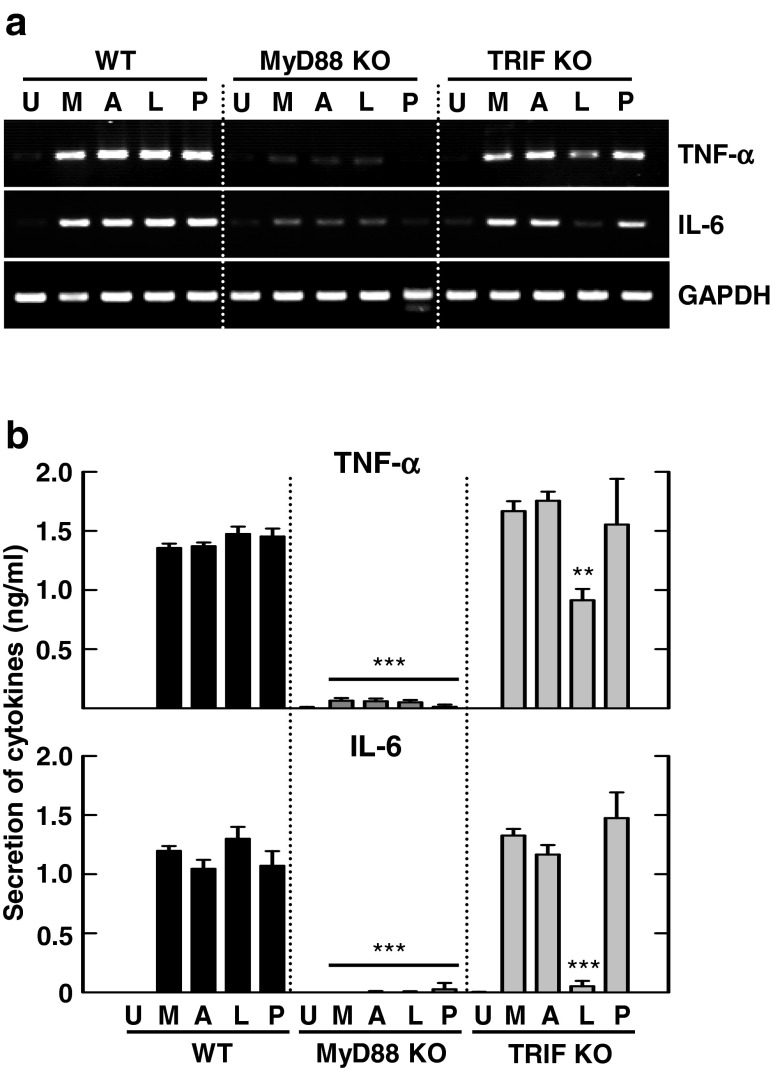
MyD88, but not TRIF, is required for Mmass-induced TNF-α and IL-6 production. **a** and **b** BMDMs from WT, MyD88 KO, and TRIF KO BMDMs were exposed to various stimulators; Mmass (MOI = 3), Mabc (MOI = 3), LPS (*L*; 100 ng/mL), or Pam3CSK4 (*P*; 100 ng/mL). **a** The cell lysates were collected and then subjected to semiquantitative RT-PCR for *TNF-α* and *IL-6*. **b** The infected supernatants were harvested and then analyzed by ELISA for TNF-α and IL-6. Data shown are representative of at least three independent experiments (expressed as means ± SD). ** *p* < 0.01, *** *p* < 0.001 (two-tailed Student’s *t*-test), compared with WT condition. *U* uninfected, *M* Mmass, *A* Mabc

In a previous study, Mabc-induced (C-C motif) ligand 2 and (C-X-C motif) ligand 2 production required the MyD88-dependent pathway [26]. However, it has not been investigated whether Mmass-induced TNF-α and IL-6 production is mediated through the MyD88 or TRIF pathway. Thus, we examined mRNA and protein expression of TNF-α and IL-6 in BMDMs from WT, MyD88 KO, and TRIF KO mice, using RT-PCR and ELISA, respectively. We found that the mRNA and protein expression of TNF-α and IL-6 was decreased significantly in MyD88-deficient BMDMs after Mmass infection, compared with the WT. In contrast, TRIF-deficient BMDMs were no different than WT cells. These data show that Mmass-induced TNF-α and IL-6 production was dependent on MyD88, but not TRIF, in BMDMs (Fig. 2a, b).

### Dectin-1 Does not Play a Role in Mmass-Induced Proinflammatory Responses in BMDMs

**Fig. 3:**
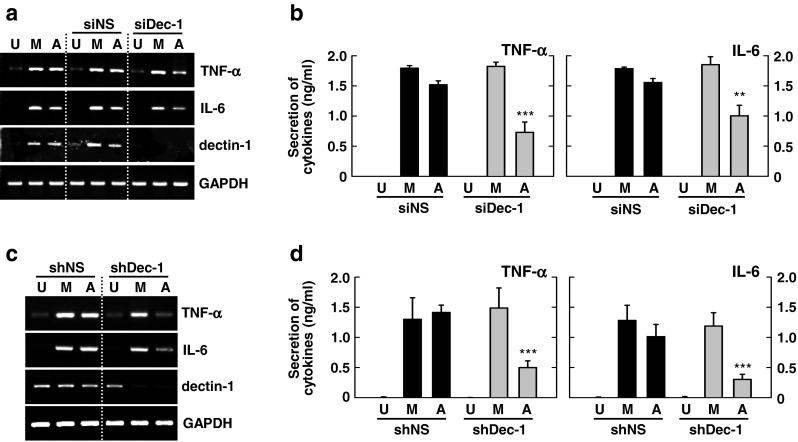
Mmass-induced TNF-α and IL-6 production is not mediated through Dectin-1. **a** and **b** Raw 264.7 cells were transfected with non-specific siRNA (siNS) or specific siRNAs against mouse dectin-1 (siDec-1) following infection with Mmass (MOI = 3) or Mabc (MOI = 3). **c** and **d** BMDMs were transduced with non-specific shRNA lentiviral particles (shNS) or lentiviral shRNA specific to dectin-1 (shDec-1), following infection with Mmass or Mabc. **a** and **c** The cell lysates were collected and then subjected to semiquantitative RT-PCR for *TNF-α*, *IL-6*, and *dectin-1*. **b** and **d** The infected supernatants were harvested and then subjected to ELISA analysis for TNF-α and IL-6. Data shown are representative of at least three independent experiments (expressed as means ± SD). ** *p* < 0.01, *** *p* < 0.001 (two-tailed Student’s *t*-test), relative to si-*NS* (**b**) or sh-*NS* (**d**). *U* uninfected, *M* Mmass, *A* Mabc

We have previously demonstrated that Dectin-1 plays a role in Mabc-mediated proinflammatory cytokine production in BMDMs [24]. To determine the role of Dectin-1 in Mmass-induced proinflammatory responses in macrophages, RAW264.7 macrophage cells were transfected with scrambled siRNA (siNS) or siRNA targeting Dectin-1 (siDec-1) prior to Mmass infection. In Dectin-1-knocked down cells, mRNA and protein synthesis of TNF-α and IL-6 was not decreased, when compared with those transfected with scrambled siRNA (siNS; Fig. 3a, b). Additionally, downregulation of Dectin-1 in BMDMs with shRNA specific to Dectin-1 (shDec-1) partially attenuated Mabc-, but not Mmass-, induced proinflammatory cytokine mRNA and protein expression (Fig. 3c, d). Consistent with our previous findings, Mabc-induced production of TNF-α and IL-6 was significantly inhibited in Dectin-1-knocked down RAW264.7 cells (Fig. 3a, b) and BMDMs (Fig. 3c, d). Together, these data suggest that Dectin-1 is not involved in Mmass-induced proinflammatory cytokine generation in macrophages.

### Mmass-Induced Proinflammatory Cytokine Generation is Mediated Through a JNK-Dependent Pathway

**Fig. 4:**
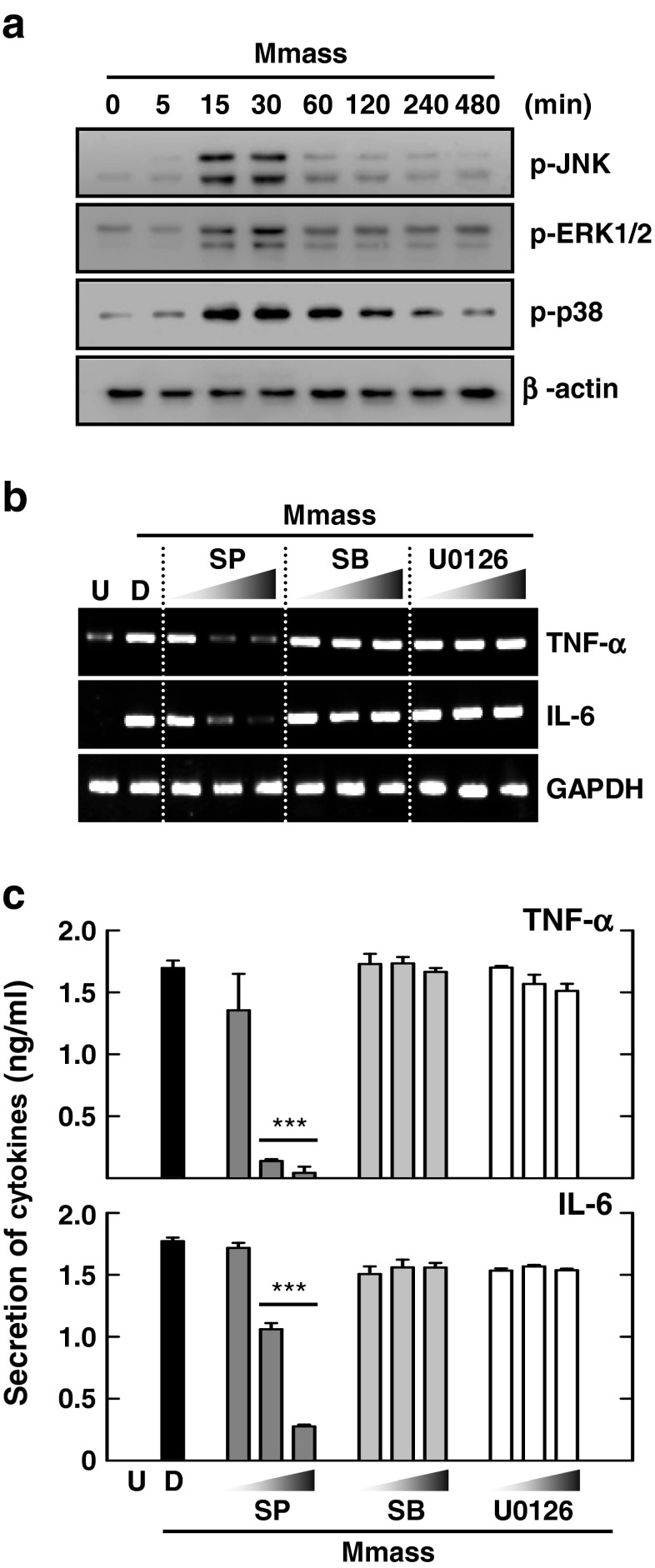
Mmass-induced inflammatory cytokine production is mediated via c-Jun N-terminal family kinases in BMDMs. **a** BMDMs were infected with Mmass (MOI = 3) for the indicated times (0-480 min). The cell lysates were collected and then subjected to Western blotting for phospho-JNK, phospho-ERK1/2, and phospho-p38. β-actin was used as a loading control. **b** and **c** BMDMs in the presence or absence of a JNK inhibitor (SP600125; 5, 20, or 30 μM), MEK-1 inhibitor (U0126; 5, 10, or 20 μM), or p38 inhibitor (SB203580; 1, 5, or 10 μM) for 45 min, before infection with Mmass. **b** The cell lysates were collected and then subjected to semiquantitative RT-PCR for *TNF-α* and *IL-6*. **c** The infected supernatants were harvested and then subjected to ELISA for TNF-α and IL-6. Data shown are representative of at least three independent experiments (expressed as means ± SD). *** *p* < 0.001 (two-tailed Student’s *t*-test), relative to the solvent control. *U* uninfected, *D* solvent control (0.05 % DMSO), *SP* SP600125, *SB* SB203580

In mycobacterial infection, MAPK activation is important for the regulation of innate immune responses and the production of various effector molecules in macrophages [19]. We showed previously that MAPK is activated and plays a role in inflammatory responses in macrophages upon Mabc infection [24]. When we infected BMDMs with Mmass, we found that Mmass robustly activated the MAPK signaling pathway (JNK, ERK1/2, and p38) in macrophages in a time-dependent manner (Fig. 4a). Consistent with previous reports [24], BMDMs infected with Mmass showed peak levels of MAPK activation at 15 and 30 min postinfection (Fig. 4a).

To investigate whether the MAPK pathway plays a role in Mmass-induced proinflammatory responses in macrophages, we pretreated macrophages with pharmacological inhibitors of the MAPK pathways and measured TNF-α and IL-6 secretion upon mycobacterial infection. As shown in Fig. 4b and c, Mmass-induced TNF-α mRNA and protein production was dose-dependently blocked in the presence of a JNK inhibitor (SP600125), but not inhibitors of the ERK1/2 (U0126) or p38 (SB203580) pathways. These data indicate that the JNK pathway is required for Mmass-induced TNF-α and IL-6 production in BMDMs.

### Mmass-Induced NF-κB Signaling is Required for Proinflammatory Cytokine Generation in BMDMs

**Fig. 5:**
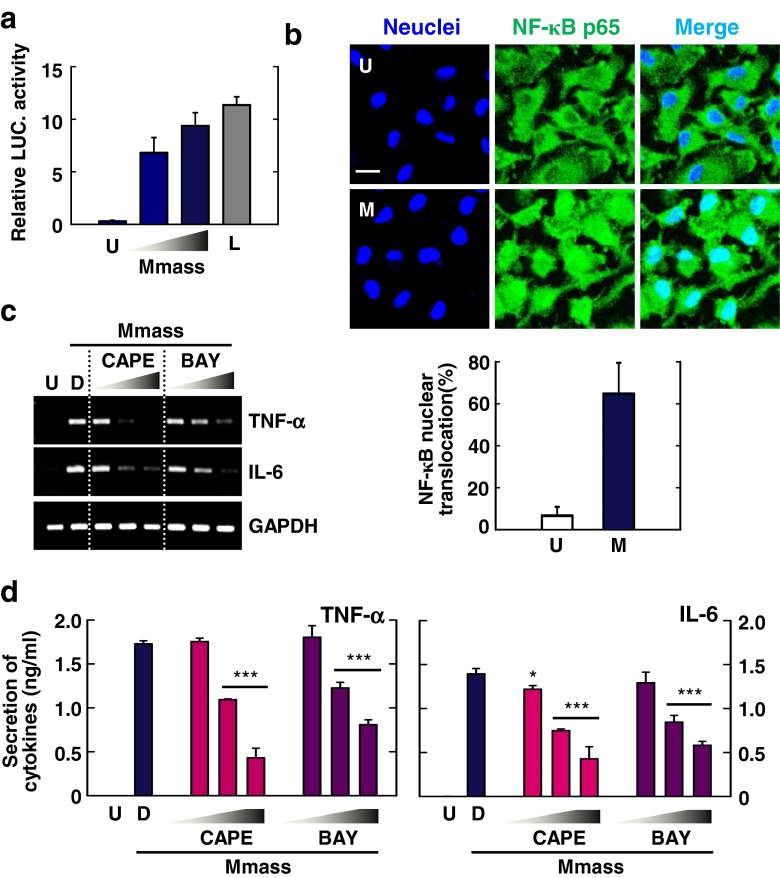
Mmass robustly increased the production of inflammatory cytokines via NF-κB activation in BMDMs. **a** BMDMs were transduced with NF-κB adenovirus luciferase construct and infected with Mmass (MOI = 1 or 3) and LPS (*L*; 100 ng/mL) for 6 h. The cell lysates were harvested and assayed for luciferase reporter activity. **b** BMDMs were infected with Mmass (M; MOI = 3) for 30 min. Then, the cells were immunolabeled with anti-NF-κB p65 antibody, anti-rabbit-AlexaFluor 488 (*green*), and DAPI to visualize the nuclei (*blue*). Representative immunofluorescence images (*upper*) and the average mean fluorescence intensity of cells exhibiting NF-κB nuclear translocation (*lower*) are shown. *Scale bars* = 20 μm. **c** and **d** BMDMs were cultured in the presence or absence of BAY 11-7082 (BAY; 0.3, 1, or 3 μM, for 45 min) or CAPE (1, 5, or 10 μM, for 2 h) before infection with Mmass. **c** Cell lysates were collected and then subjected to semiquantitative RT-PCR for *TNF-α* and *IL-6*. **d** The infected supernatants were harvested and then subjected to ELISA for TNF-α and IL-6. Data shown are representative of at least three independent experiments (expressed as means ± SD). * *p* < 0.05, *** *p* < 0.001 (two-tailed Student’s *t*-test), relative to the solvent control. *U* uninfected, *D* solvent control (0.05 % DMSO)

In mycobacterial infection, the NF-κB signaling pathway plays a key role in the induction of inflammatory cytokine generation and antimicrobial protein synthesis in TLR-triggered innate immune responses [18, 27]. To determine whether Mmass activated the NF-κB signaling pathway, we performed a NF-κB luciferase assay in BMDMs transduced with adenovirus encoding a luciferase reporter plasmid containing response elements for NF-κB (Ad-NF-κB-Luc). As shown in Fig. 5a, Mmass infection strongly increased NF-κB reporter gene activities in BMDMs transduced with Ad-NF-κB-Luc, in a multiplicity of infection (MOI)-dependent manner. There was no significant increase in NF-κB promoter activities in BMDMs transduced with control adenovirus (data not shown). We also determined that Mmass infection stimulated the translocation of NF-κB p65 from the cytoplasm to the nucleus, as assessed using confocal microscopy after 30 min of infection (Fig. 5b).

We next examined whether NF-κB signaling was involved in the induction of mRNA expression of TNF-α and IL-6 in response to Mmass. As shown in Fig. 5c and d, Mmass-induced TNF-α and IL-6 production was dose-dependently abrogated in BMDMs by pretreatment with Bay 11-7082 (BAY) or caffeic acid phenethyl ester (CAPE), specific inhibitors of the NF-κB signaling pathway. From these results, Mmass-induced NF-κB signaling activation is required for proinflammatory cytokine generation in BMDMs.

### Mmass-Induced Proinflammatory Cytokine Generation is Dependent on NOX2-Dependent ROS Generation in BMDMs

**Fig. 6:**
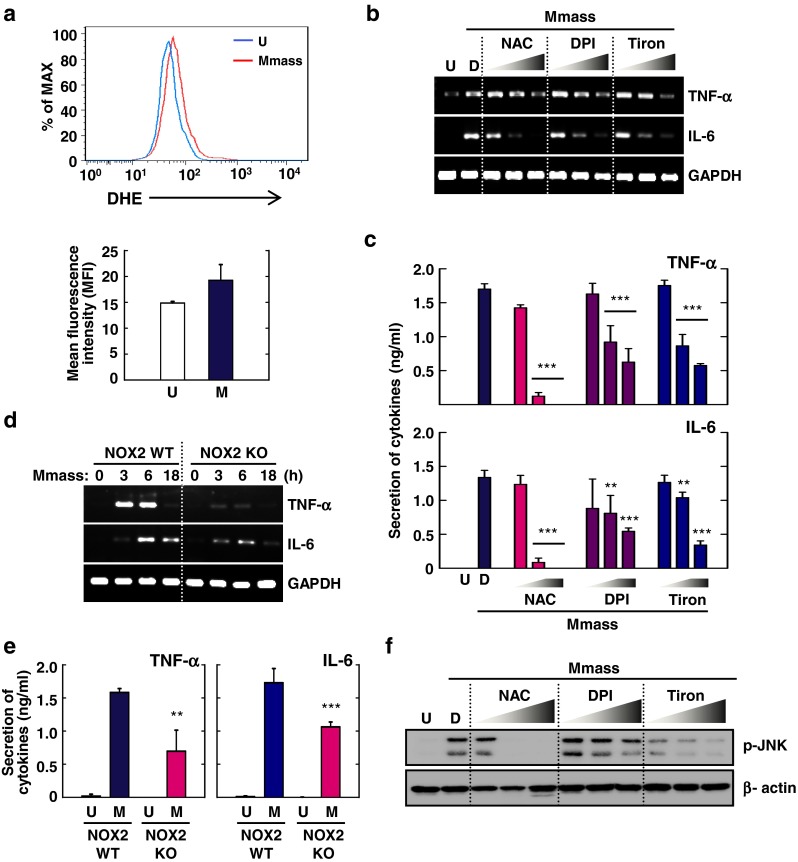
Intracellular ROS generation is involved in Mmass-induced cytokine production. **a** FACS analysis of BMDMs were infected with Mmass (MOI = 3) for 30 min, and stained with DHE for 30 min. Representative images (*upper*) and quantitative analysis of mean fluorescence intensities (*lower*) are shown. **b**, **c**, and **f** BMDMs were cultured in the presence or absence of an antioxidant (NAC; 10, 20, or 30 mM), a NADPH oxidase inhibitor (DPI; 1, 5, or 10 μM), or a superoxide scavenger Tiron (10, 20, or 30 mM) for 45 min, before infection with Mmass. **d** and **e** BMDMs from NOX2 WT and NOX2 KO BMDMs were infected with Mmass (M) for the indicated times (0-18 h). **b** and **d** Cell lysates were collected and then subjected to semiquantitative RT-PCR for *TNF-α* and *IL-6.* **c** and **e** The infected supernatants were harvested and then subjected to ELISA analysis for TNF-α and IL-6. **f** Cell lysates were collected and then analyzed by Western blotting for phospho-JNK; β-actin was used as a loading control. Data shown are representative of at least three independent experiments (expressed as means ± SD). ** *p* < 0.01, *** *p* < 0.001 (two-tailed Student’s *t*-test), compared with the solvent control (**c**) or NOX2 WT (**e**). *U* uninfected, *D* solvent control (0.05 % DMSO)

Previous studies suggested that ROS generation played multiple roles, in mycobacterial killing, regulation of cell death, cytokine production, and antimicrobial protein activation, in TLR-triggered innate immune responses [22, 28]. We first examined whether Mmass induced BMDMs using the oxidative fluorescent dye dihydroethidium (DHE) to determine intracellular superoxide generation. As shown in Fig. 6a, Mmass-induced superoxide production was detected rapidly. We next examined whether infection with Mmass-dependent ROS generation was required for cytokine expression and production. Treatment with various antioxidants, such the general ROS scavenger NAC, the NADPH oxidase inhibitor DPI, and the superoxide scavenger Tiron, dose-dependently decreased Mmass-induced mRNA expression and protein production of TNF-α and IL-6 in BMDMs (Fig. 6b, c). Previously, it has been shown that mycobacterial infection BMDMs involves significant levels of NOX2 expression [22]. Thus, we further investigated the role of NOX2 in infection and Mmass-mediated cytokine production. Mmass-induced mRNA expression and production of TNF-α and IL-6 in BMDMs from NOX2 KO mice were nearly abolished, compared with WT controls (Fig. 6d, e). We next examined the interaction between the JNK and ROS signaling pathways. BMDMs were pre-treated with antioxidants followed by Mmass stimulation. We found that pre-treatment with ROS blockers significantly decreased JNK phosphorylation in a dose-dependent manner (Fig. 6f). These findings suggest that NOX2-dependent generation of ROS is involved in Mmass-mediated inflammatory cytokine induction and JNK signaling in infection in murine macrophages.

### R Morphotypes of Mmass Elicit Higher Production of Proinflammatory Cytokines and JNK Activation Than S Morphotypes

**Fig. 7:**
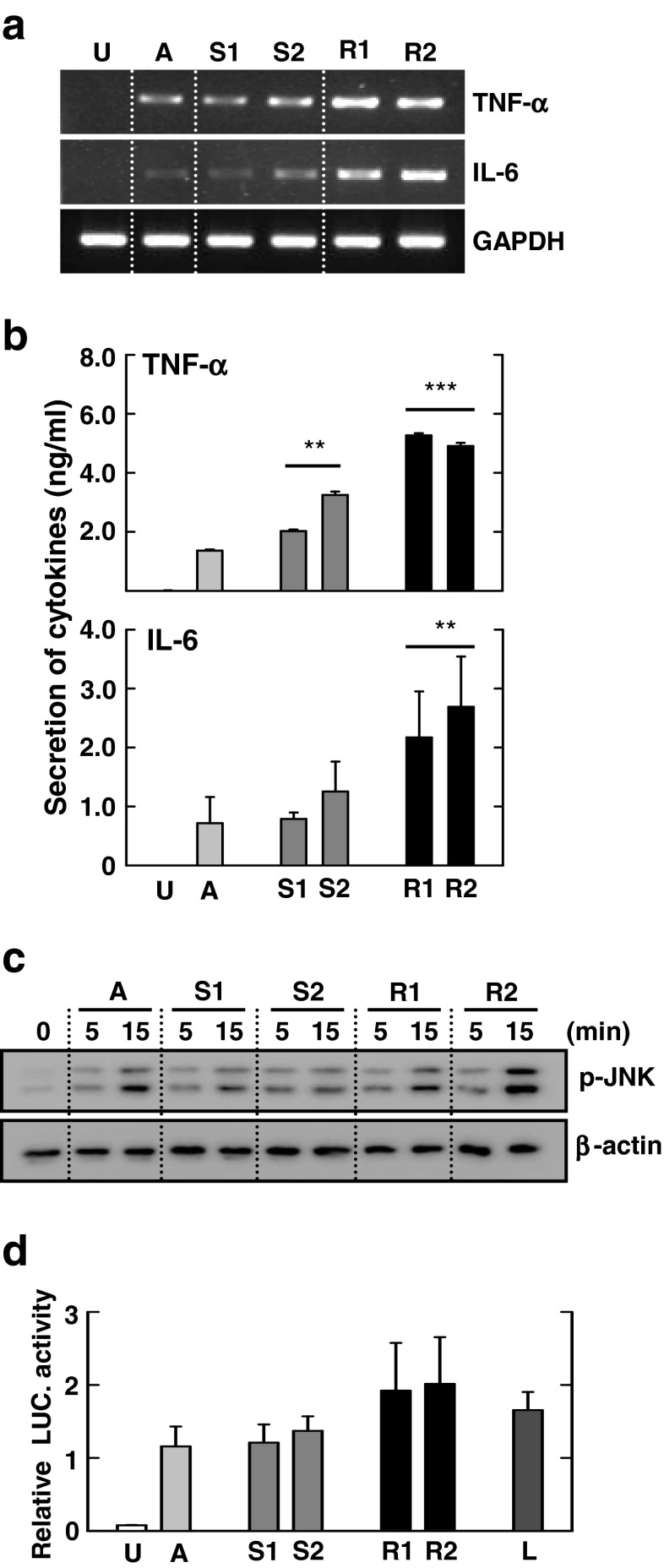
Inflammatory expression is upregulated in rough-type clinical strain-infected murine macrophages. **a**–**d** BMDMs were infected with type strain A (MOI = 3), smooth-type clinical strains (*S1* and *S2*; MOI = 3) and rough-type clinical strains (*R1* and *R2*; MOI = 3). **a** The cell lysates were collected and then subjected to semiquantitative RT-PCR for *TNF-α* and *IL-6*. **b** The infected supernatants were harvested and then subjected to ELISA analysis for TNF-α and IL-6. **c** BMDMs were infected with Mmass strains for the indicated times (5 and 15 min). Cell lysates were collected and then subjected to Western blotting for phospho-JNK. β-actin was used as a loading control. **d** BMDMs were transduced with NF-κB adenovirus luciferase construct and infected with each type of Mmass or LPS (*L*; 100 ng/mL). Cell lysates were harvested and assayed for luciferase reporter activity. Data shown are representative of at least three independent experiments (expressed as means ± SD). ** *p* < 0.01, *** *p* < 0.001 (two-tailed Student’s *t*-test), relative to Mmass type strain infection. *U* uninfected, *A* type strain CIP 108297, *S1* smooth-type clinical strain KMRC00136-13008, *S2* smooth-type clinical strain KMRC00136-13011, *R1* rough-type clinical strain KMRC00136-13009, *R2* rough-type clinical strain KMRC00136-13012

Previous studies showed that Mabc R variants were associated with TLR2-dependent hyper-proinflammatory responses via increased synthesis/exposure at the cell surface of lipoproteins [29]. However, the association between bacterial colony morphotypes and innate immune responses in Mmass infection has not been characterized. To examine whether there was any relationship between morphotypes of Mmass and inflammatory responses in macrophages, we infected murine BMDMs with four strains: two smooth (S1 and S2) and two rough types (R1 and R2). As shown in Fig. 7a and b, the mRNA and protein expression of TNF-α and IL-6 were increased significantly in BMDM after Mmass R morphotype infection, compared with those with S morphotype infection. When phospho-JNK levels were compared among BMDMs infected with various clinical strains, the R morphotype induced higher activation of JNK than S morphotype in BMDMs (Fig. 7c). Moreover, the NF-κB luciferase assay showed that the R morphotypes of Mmass induce higher levels of luciferase activity than S morphotypes (Fig. 7d). These data show that the R morphotypes of Mmass induced greater proinflammatory cytokine release along with increased activation of JNK and NF-κB signaling pathways compared with the S morphotypes.

## Discussion

Mabc complex, the second most common cause of atypical mycobacterial infection following *M. avium*-*intracellulare* complex, is the most virulent RGM. These infections are frequently associated with infectious diseases affecting the lungs, skin, or soft tissues [30]. In recent years, the members of Mabc complex, Mmass and *M. bolletii* were identified from other RGM species through genetic investigations. The development of 16S rRNA gene sequence analysis and the sequencing of rpoB, sodA, and hsp65 genes of NTM resulted in species-specific identification of Mmass [31]. Moreover, recent studies have shown that mycobacterial genotyping between Mabc and Mmass shows a significant association of specific genotypes with clinical phenotypes and disease progression [32]. Inflammatory responses play important roles in host defense and in dictating immunopathogenesis during mycobacterial infection [33, 34]. Bacterial growth and infection-induced mortality were significantly increased in a TNF-deficient mouse model of Mabc infection; however, these findings were not observed with the subspecies *M. chelonae* [15]. Although there has been significant progress in identifying Mmass from other Mabc complex species, little is known about the host inflammatory responses to Mmass.

Compared with tuberculosis, much less is known about the roles of TLRs in atypical mycobacterial infection. Our study showed that Mmass robustly activated macrophage inflammatory responses through TLR2 and MyD88, but not Dectin-1. Earlier studies showed that lipomannans purified from *M. chelonae* and a clinical strain of *M. kansasii* induced mRNA expression and secretion of TNF-α and IL-8 from human THP-1 cells by a TLR2-dependent mechanism [35]. Mabc-mediated TNF-α production is abrogated in TLR2- or MyD88-deficient BMDMs, reinforcing a critical role for TLR2-MyD88 in Mabc-induced macrophage activation [36]. Our previous studies also showed that TLR2 and Dectin-1 are involved in the production of proinflammatory cytokines and chemokines to *M. ulcerans* or Mabc in human keratinocytes and murine macrophages, respectively [24, 37]. Dectin-1 is a type II transmembrane non-opsonic receptor and plays a key role in the binding and response to fungal-derived β-1,3 and β-1,6 glucan particles [38, 39]. It is expressed on most immune cells involved in innate immunity and plays an essential role in phagocytosis and antifungal activities in macrophages [38, 39]. In mycobacterial infections, signaling through Dectin-1 is required for the production of several cytokines, including IL-6, RANTES (regulated on activation, normal T expressed, and secreted), and granulocyte colony-stimulating factor by macrophages [40]. Importantly, Mabc infection leads to activation of the NLRP3 inflammasome and caspase-1 cleavage in human monocyte-derived macrophages via Dectin-1 [23].

Mmass-induced JNK and NF-κB signaling pathways play an essential role in the production of TNF-α and IL-6 in BMDMs. After engagement of innate immune receptors by mycobacterial strains, the intracellular signaling pathways, including the MAPK family and NF-κB signaling, lead to activation of the synthesis of innate effectors and inflammatory mediators in mononuclear phagocytes [16, 18, 19]. Recent studies have shown that monocytes from patients with Mabc lung infection have decreased MAPK activity and production of proinflammatory cytokines, including TNF-α and IL-6 [41]. However, Mabc induces higher production of proinflammatory cytokines and chemokines through the TLR2 and MAPK pathways than *M. avium* [36]. In human cells, Mabc-induced TNF-α production depends on the activation of the ERK1/2 and p38 MAPK pathways [36]. Further, *M. avium* infection prior to BCG resulted in the production of IL-10 in dendritic cells through the TLR2-p38 MAPK signaling pathway [42]. Our data are unique in demonstrating the critical role of JNK signaling in proinflammatory responses to Mmass in macrophage infection. Mmass-induced ROS generation was required for proinflammatory cytokine responses in macrophages. In a previous study we found that the ROS production played an essential role in autophagy and proinflammatory cytokine production in Mtb-eis-infected BMDMs [28]. We have recently shown that Mabc infection of macrophages leads to the induction of CCL2 through ROS-mediated NF-κB signaling activation [26]. Cellular ROS are required for the cutaneous innate immune responses, through inhibition of intracellular *M. ulcerans* growth in keratinocytes [37]. These findings suggest a novel role for ROS signaling in the activation of macrophage inflammatory responses, in particular, by activating JNK-dependent mechanisms in infected macrophages.

To our knowledge, this is the first report that S morphotypes of Mmass elicit less profound effects on innate immune signaling through JNK activation in macrophages. A variety of clinical strains of atypical mycobacteria show diversity in colony morphology: R and S colony phenotypes among NTM strains [43]. A recent study involving genome- and transcriptome-wide analyses identified newly defined genetic lesions responsible for glycopeptidolipids, which control switching between the R and S morphologies in Mabc [44]. R variants of Mabc markedly activate the innate immune response through TLR2, while S variants do not, partly due to the deficiency of cell wall glycopeptidolipids, which masks underlying cell wall lipid components involved in induction of inflammatory responses [45]. Moreover, the R variant of Mabc infection led to hyperlethality in mice [46] and was found to be associated with severe clinical phenotypes characterized by hyper-inflammatory responses [29]. Interestingly, Mabc isolated from the upper lobe fibrocavitary form of pulmonary Mabc infection was associated with stronger inflammatory responses and increased bacterial loads in the lungs of infected mice compared to the nodular bronchiectatic form of disease [47]. However, previous studies reported no significant difference in human cell secretion of TNF-α between infection with R and S isolates of Mabc or *M. avium* [36]. Differences in the inflammatory responses induced by each Mabc subspecies, along with the clinical phenotypes associated with each subspecies, are beginning to be recognized. Additional studies clarifying the relationship between Mmass-induced inflammatory responses and clinical manifestations of infection are urgently needed. Taken together, our data suggest that Mmass activates macrophage innate immune responses through TLR2-dependent JNK, NF-κB, and ROS signaling pathways. These in vitro effects may be associated with the inflammatory responses and clinical features, and may support the development of new therapeutics for Mmass-mediated atypical mycobacterial infection.
